# Population Structure and Regeneration Status of Woody Plant Species in Tulu Korma Dry Afromontane Forest, West Shewa Zone, Oromia, Ethiopia

**DOI:** 10.1155/2023/9964663

**Published:** 2023-04-20

**Authors:** Diriba Deressa, Meseret C. Egigu, J. M. Sasikumar

**Affiliations:** Haramaya University, School of Biological Sciences and Biotechnology, P.O. 138, Dire Dawa, Ethiopia

## Abstract

A study was conducted on *Tulu Korma* afromontane forest to assess woody plant species' population structure and natural regeneration status. Data were collected from 52 main quadrats of 400 m^2^ for mature woody species and 260 subplots of 25 m^2^ for seedlings and saplings. All live woody plant species were recorded with their densities, heights, and DBH. Frequency, basal area, importance value, and Shannon–Wiener diversity indices were computed. A total of 101 species that belonged to 45 families were documented. Diversity and evenness indices were 3.44 and 0.7, respectively. Combined density of woody species of all developmental stages was 4971 stems ha^−1^ of which 39, 32, and 28% were with DBH < 3.5 cm (seedlings), between 2 and 10 cm (saplings), and >10 cm (mature wordy species), respectively. The total basal area of individuals with DBH ≥ 3.5 cm was 116.18 m^2^ ha^−1^. *Olea europaea* and *Podocarpus falcatus* were the most dominant species. About 41.58% of the species had IVI < 1. Population structure based on combined densities revealed that density of seedling > sapling > mature individuals, suggesting healthy population structure and good regeneration. On individual basis, however, species showed different patterns of population structure of which 12, 51, and 37% species showed good, fair, and poor regenerations, respectively. Species with least IVI and poor regeneration should be given conservation priority.

## 1. Introduction

Ethiopia, located in the tropics, has a wide range of ecological niches arising from varying topography and climate that created environments conducive for the establishment of various forms of life [1]. The altitudes of the country range from 110 meters below sea level in the Dalol (Afar) depression to the highest mountain (4,620 meters above sea level), Ras Dejen in the Semen Mountains [2]. These diverse ecological and habitats make Ethiopia one of the floristically rich tropical countries with estimated vascular plant species number between 6500 and 7000. Of these plant species, about 12 to 19% are endemic [3]. Ethiopia is not only wild floras diverse but also a very important center of crop genetic diversity and considered as one of the twelve Vavilovian centers of diversity [4].

However, this tremendous wealth of natural resources and biological diversities of the country has been facing serious conservation challenges. For example, the forest cover, which was about 35% of the total landmass of the country a hundred years ago, has currently been decreased to less than 3% [5]. In Ethiopia, forests are sources of livelihood for millions of people. The main driver for forest degradation is high rate (∼3% per annum) of human population growth [6] that causes human-based deforestation through different activities in order to meet their livelihood. Increase in human population subjected forests to extensive forest clearing for agricultural land expansion, resettlement, overgrazing, fuel wood, fodder, and construction materials [7, 8]. Deforestation of natural forest has detrimental effects including direct loss of biodiversity, changes in ecosystem structure and function that uphold relevant ecosystem services, and negatively affects the socio-economic development and welfare of human-beings [9, 10].

In order to prevent forest degradation and biodiversity loss, appropriate management strategies that help in the maintenance of the existing forest status and restoration of deforested lands are crucial. For this, however, the diversity, population structure, and species regeneration status of the forest is a requirement. Knowledge on population structure based on the number of seedlings, saplings, and mature individual plants; for example, helps to determine the regeneration status of individual species of a given plant community.

In Ethiopia ecological studies that dealt with woody species diversity, population structure and regeneration status were conducted in different dry afromontane forests found in different regions [11–25]. Tulu Korma Forest is one of the dry afromontane forests of the country found in west central highlands of Ethiopia. Although a study on its floristic composition and community types was conducted by Zewdie et al. [26], population structure and regeneration status of woody species of this vegetation is lacking. Here, we hypothesized that the regeneration status of the vegetation in general is of poor condition due to human interference as happening elsewhere in similar vegetation in Ethiopia. This study was, therefore, designed to assess population structure and regeneration status of woody plant species of this forest to help formulate appropriate management practices.

## 2. Materials and Methods

### 2.1. Description of the Study Area and Sampling Design

**Figure 1:**
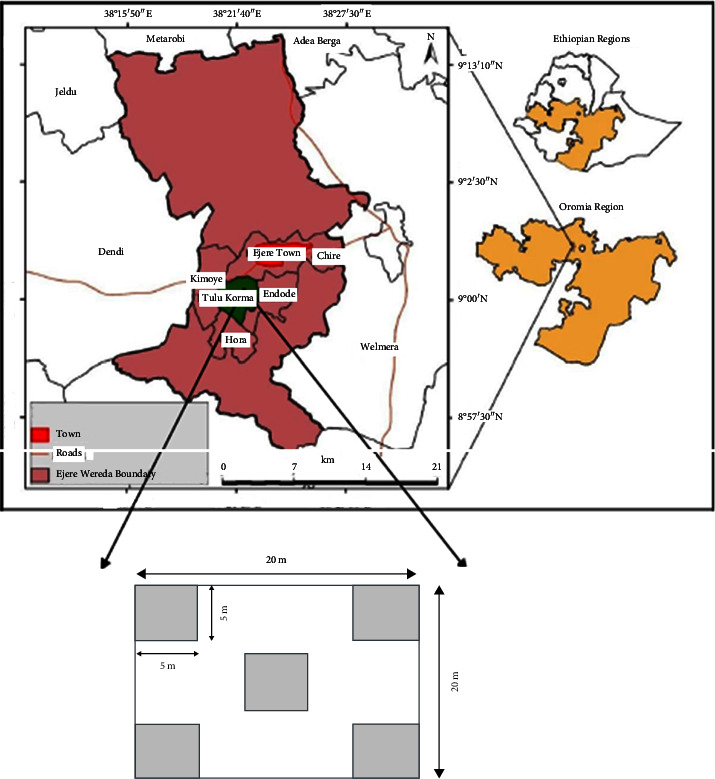
Location map of Tulu Korma Forest and Ejere areas with sampling plot design.

Tulu Korma forest (09°01.188′ N and 038°21.570′ *E*) is one of the dry afromontane forests found in West Shewa zone of Oromia regional state, Ethiopia (Figure 1). This natural and plantation mixed forest is located in the central part of the country 55 km away from Addis Ababa to the west within altitudinal range of 2,163−2,267 m above sea level. The Tulu Korma forest area is generally characterized by diverse topographic conditions. There are escarpments, hills, gorges, and flat to moderately sloping plateau, which is dissected by deep gullies, bordered by river valleys, and other landforms. The diversity of terrain of this area contributes to the slight variation of natural vegetation by determining local variations in climate and soil composition, which enabled the area to have diverse floristic composition [27]. The mean annual temperature and rainfall are 17°C and 1119 mm, respectively (National Meteorology Service Agency).

**Figure 2:**
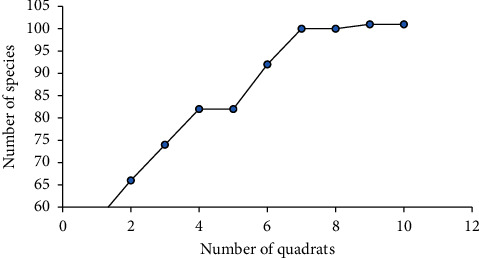
Species accumulation curve for sample size determination.

Reconnaissance survey was made in the study area in order to have general setting of the environment so as to estimate the position, number and length of transects to be laid across the forest. Following a reconnaissance survey, five line transects (1 to 1.2 km long) that were far apart from each other by at least 500 m were laid across the sampling area along different directions to purposively cut across the entire vegetation components. Along transect, the first sample plot was taken randomly and the subsequent plots were laid down systematically at 100 m intervals. From each transect at least 10 sample plots were sampled. Totally, 52 main sample plots of size 20 × 20 m (for mature woody species) that had five 5 × 5 m per main plot for saplings and seedlings data were sampled (Figure 1). The total number of sample plots was derived from species accumulation curve plotted using number of sample plots (*X*-axis) against number of species encountered (*Y*-axis) on each transect (Figure 2).

### 2.2. Vegetation Data Collection

For vegetation data collection, all live woody species (WS) in each sampling plot were considered. All mature woody species rooted within the main plot, and saplings and seedlings of woody species within the subplots of 5 × 5 m were identified and recorded. Mature individuals are those with diameter at breast height [DBH], (measured at 1.3 m above the ground) of >10 cm. Individuals with DBH range of 3.5 to 10 cm were considered as sapling while individuals with DBH < 3.5 cm were considered as seedlings [28]. Density and stem diameter at breast height were recorded for all woody species with stem diameter ≥ 3.5 cm. DBH was measured using diameter tape. When a stem was found branching at around the breast height, the DBH was measured separately for each branch and average DBH was taken. For those woody species having DBH < 3.5 cm, only their densities were recorded. All encountered WS were then preliminarily identified in the field by using the available literature (different volumes of flora of Ethiopia and Eritrea). Voucher specimens were then collected, dried, and pressed for further identification and confirmation of the authenticity in Haramaya University Herbarium. Furthermore, the scientific names of the collected plants were verified by consulting “the plants of the world online” (https://powo.science.kew.org/). For future reference, the identified plants were then deposited in Haramaya University Herbarium, Ethiopia.

### 2.3. Data Analysis

#### 2.3.1. Structural Data Analysis

Density and basal area or dominance (BA or Do) were considered as main structural data. Density, which is a count of stems of each woody species including mature ones, saplings, and seedlings from all sample plots were expressed as number of stems per hectare. Basal area defined as a cross-sectional area of a stem at 1.3 m for individual species with DBH ≥ 3.5 cm was computed from DBH value and converted to m^2^ ha^−1^. It was calculated using equation (1) as follows:

(1) $$\begin{array}{c} BA=\pi \frac{d^{2}}{4}\text{,} \end{array}$$

where BA = Basal area in m^2^ ha^−1^, *d* = diameter at breast height and *π*=3.14159265.

The forest structure was further described using parameters including frequency (the proportion of sample plots in which a given species was observed from the total sample plots), relative density (RD), relative frequency (RFr), relative dominance (RDo), and important value index (IVI). The latter four parameters were calculated using equations (2)–(5), respectively.

(2) $$\begin{array}{c} RD=\frac{Density\;of\;species\;A}{Total\;density\;of\;all\;species}\;X\;100, \end{array}$$

(3) $$\begin{array}{c} RFr=\frac{frequency\;of\;species\;A}{Total\;frequency\;of\;all\;species}\;X\;100\text{,} \end{array}$$

(4) $$\begin{array}{c} RDo=\frac{Dominance\;of\;species\;A}{Total\;dominance\;of\;all\;species}\;X\;100\text{,} \end{array}$$

(5) $$\begin{array}{c} IVI=RD+Rfr+RDo\text{.} \end{array}$$

Shannon–Wiener diversity (*H*′) and Equitability or Evenness indices (*E*) were computed to determine the diversity and evenness of the woody species using equations (6) and (7), respectively.

(6) $$\begin{array}{c} H\prime =-\overset{s}{\underset{i=1}{\sum }}p_{i}\ln p_{i,} \end{array}$$

where *H*′ = Shannon diversity index, *S* = the number of species, *p*_*i*_ = the proportion of individuals of the ith species expressed as a proportion of total number in the sample, and ln = the natural logarithm.

(7) $$\begin{array}{c} E=\frac{H\prime }{H\;\max}=\frac{H\prime }{lnS}\text{,} \end{array}$$

where *E* = Evenness; *H*′ = Shannon–Wiener Diversity Index; *H*max = ln (*S*); *S* = total number of species in the sample.

#### 2.3.2. Analysis of Population Structure and Regeneration Status

The population structures of individual and all species combined were shown using histograms based on densities of seedlings, saplings, and mature woody plant species. From the appearance of population structure, regeneration status of each woody species and all species combined was explained [28, 29]. Regeneration status of a species was considered ‘good' when number of seedlings is > saplings > mature; fair when seedlings > or ≤ saplings ≤ mature; poor when a species is represented by sapling only (saplings may be ≤ or ≥ mature) [20].

## 3. Results

### 3.1. Species Richness and Diversity of Woody Plant Species

In this study, 101 species belonging to 79 genera and 45 families were documented (Table 1). Top five species rich families were Fabaceae, Asteraceae, Euphorbiaceae, Moraceae, and Celastraceae (Table 1). The Fabaceae constituted about 17% of the total species recorded followed by Asteraceae (ca. 6%); Euphorbiaceae (ca. 5%); Moraceae, Celastraceae, Myrtaceae, and Rubiaceae (ca. 4% each). Rutaceae, Lamiaceae, Anacardiaceae, and Myrsinaceae were represented by 3 species each, whereas Verbenaceae, Solanaceae, Acanthaceae, Rhamnaceae, Cupressaceae, Sapindaceae, Rosaceae, and Flacourtiaceae were represented by 2 species each. The rest 25 families were represented by one species each. Shannon–Wiener Diversity (*H′*) and Equitability (*E*) indices were 3.44 and 0.7, respectively.

### 3.2. Density, Frequency, Dominance, and Important Value Index

The density of individual species ranged from 1.0 to 247 stems ha^−1^. The density of all woody species combined was 4971 stems ha^−1^. *Olea europaea*, *Juniperus procera*, *Podocarpus falcatus*, *Acacia abyssinica*, *Ficus palmata,* and *Cordia africana* were top six densest species and accounted for about 35% of woody species with DBH value of ≥3.5 cm (Table 1). Frequency of individual species ranged from 4 to 67%. The most frequent species were *Podocarpus falcatus* followed by *Olea europaea*, *Acacia abyssinica*, *Carissa spinarum*, *Ficus sur*, *Cordia africana*, *Croton macrostachyus*, *Dovyalis abyssinica,* and *Juniperus procera* (Table 1). Basal area (dominance) was computed for individuals with DBH ≥ 3.5 cm and the basal area of the whole species combined was 116.18 m^2^ ha^−1^. *Olea europaea*, *Podocarpus falcatus*, *Acacia abyssinica,* and *Juniperus procera* were the most dominant species. Together their relative dominance comprised 71.41% of the total basal area. Computation of IVI showed that *Olea europaea* and *Podocarpus falcatus* were the two most important species that were most abundant, frequent, and dominant. Together they contributed 22.87% of the total IVI. About 41.58% of the species had IVI less than one and appeared to be least frequent, abundant, and dominant (Table 1).

### 3.3. Population Structure and Regeneration Status

**Figure 3:**
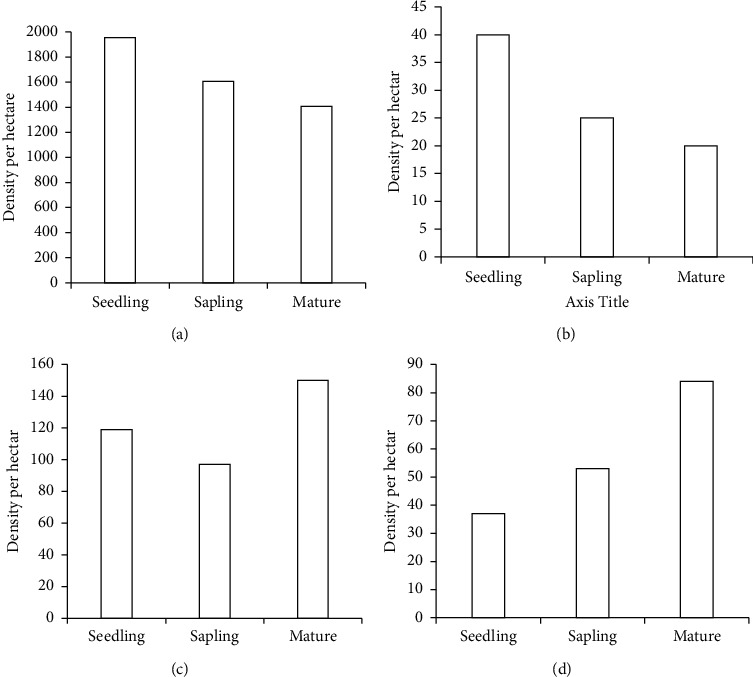
Representative population structure patterns of woody plant species in Tulu Korma afromontane forest based on densities of seedlings, saplings, and mature individuals. Note: (a) is for all species combined and suggests good regeneration, (b) is representative species with good regeneration, (c) is representative species with fair regeneration, and (d) is representative species tending to have poor regeneration.

In order to visualize the population structure of this forest, we categorized woody species into three girth classes based on their DBH measurement. That is, individuals with DBH < 3.5 cm (seedlings); individuals with DBH from 3.5 to 10 cm (saplings) and individuals with DBH > 10 cm representing mature woody species. A histogram of density (*Y*-axis) vs DBH class (on *X*-axis) was drawn to reveal population structure of the entire species combined (Figure 3(a)). Furthermore, densities of individual species at different developmental stages were analyzed to roughly visualize their population structure patterns and regeneration status. The result showed that 12, 51 and 37% of the species had good (represented by Figure 3(b)), fair (represented by Figure 3(c)), and poor (represented by Figure 3(d)) regeneration status, respectively.

## 4. Discussion

### 4.1. Species Richness and Diversity

Determination of the population structure and natural regeneration status of a forest is crucial to designing of sound conservation strategies. In this study, we documented 101 woody plant species distributed in 45 families. The Shannon–Wiener diversity index (*H*′) and evenness values of this forest were 3.44 and 0.7, respectively. The value of Shannon–Wiener diversity index usually found to fall between 1.5 and 3.5, and rarely surpasses 4.5 [30]. The Shannon diversity index value observed in Tulu Korma Forest fall within the range (0.70–3.57) reported for other dry forests of the Subsaharan region [31, 32]. Based on this assumption, the diversity index obtained for this forest shows that Tulu Korma Forest has high diversity with different species having more or less uniform abundance. In this study, we used species accumulation curve (Figure 2) to determine the number of sample plots required. Comparing with many other similar studies that followed the same procedure, Tulu Korma was more species rich and diverse in woody plants than, for example, Wof Washa natural forest in north-east Ethiopia with 62 woody species [17]; Gichi *In-situ* Forest conservation site, north-western Ethiopia with 47 species [22]; Gelawoldie community forest, north-western Ethiopia with 59 species [25]; Denkoro Forest with 64 species [13], and Angada forest with 87 species [33]. Variation between sites in terms of species richness and diversity may be attributed to their difference in altitude, climate, edaphic condition, and/or biotic effects including human disturbance [24, 29]. High species richness and diversity of Tulu Korma Forest suggest the need to paying attention in terms of protecting the area from future possible destruction so that a few remnant montane forests of the country are conserved.

In this study, variation in familial species richness was observed. For example, Fabaceae was the most species rich family. Although some similar studies conducted in different parts of Ethiopia report families other than Fabaceae as a species rich family [21, 24] (e.g., Rosaceae by [21]; Asteraceae by [24]), Fabaceae was previously reported as the most species rich family by different researchers e.g., [12, 18, 20, 23, 25, 34, 35] in several other similar vegetation of the country. The dominance of Fabaceae or other family in terms of number of species may be ascribed to its successful dispersal strategy and adaptation to a wide range of ecological conditions. Success in dispersal and adaptation to varying environments in turn is directly related to the number of growth forms (herbaceous to woody) within a family that aid to encompass morphologically differentiated groups to fit to varying dispersal and pollination modes, hence, species proliferation within a family [36].

### 4.2. Density, Frequency, Dominance, and Importance Value Index

The density of all species combined with DBH ≥ 3.5 cm was 4971 stems ha^−1^. Top known densest indigenous woody plant species in a decreasing order were *Olea europaea*, *Juniperus procera*, *Podocarpus falcatus*, *Acacia abyssinica*, *Ficus palmate,* and *Cordia africana*, and they had cumulative percentage of 34.14. These species including others such as *Carissa spinarum*, *Ficus sur,* and *Dovyalis abyssinica* were also the most frequent species. Combined density of woody plant species reported in this study was greater than most results, for example, those reported by Yineger et al. [37] (898 stems ha^−1^) and [17] (698 stems ha^−1^) from other similar afromontane forests of the country. Most probably, the relatively higher total density of woody plant species in Tulu Korma Forest is related to the higher species number recorded. Differences in total density among sites may also be attributed to the difference in the extent of anthropogenic pressure between sites. Majority of species (ca. 60%) including *Dicrostachys cinerea*, *Euclea divinorum*, *Ficus vasta, Grewia ferruginea*, *Ocimum lamiifolium*, *Ocimum urticifolia,* and *Pterolobium stellatum* had low (<20%) frequency. Variations in density and frequency among individuals in the same site may be ascribed to heterogeneity of the environment in terms of microsite conditions that determine occurrence and reproduction. In this study, about 41.58% of the species (indicated in Table 1) had IVI less than one and appeared to be least frequent, abundant and dominant. According to Neelo et al. [29] ecologically significant species of an ecosystem are implied by their IVI values. The IVI value also indicates how much emphasis should be given to a species for conservation [11].

### 4.3. Population Structure and Regeneration Status

Combined density of all species demonstrated that number of seedlings > saplings > mature woody species. Such population structure is explained as having good regeneration status. Several authors [14, 15, 22, 24, 25, 38] interpret that a forest with such a pattern is a healthy forest that actively regenerates with naturally ongoing new individual recruitment. However, there is a reservation to generalize that such pattern of population structure is always an indicative of good regeneration, because it is based on an assumption that there is equal mortality among size (DBH) classes, which may not always be true biologically [39]. On individual basis, 11.88% of the total species including *Acacia abyssinica*, *Croton macrostachyus*, *Milletia ferruginea*, *Syzygium guineense*, *Sesbania sesban, Allophylus abyssinicus*, *Apodytes dimidiata*, *Calpurnia aurea*, *Sapium ellipticum*, *Stephania abyssinica*, *Tacazzea apiculata,* and *Vernonia urticifolia* showed similar patterns of population structure. This shows that they are in a healthy regeneration status. About 51% of the total species including *Olea europaea*, *Podocarpus falcatus*, *Acacia abyssinica*, *Juniperus procera*, *Carissa spinarum,* and *Croton macrostachyus* that appeared more frequent, dense and dominant had fair regeneration status. However, 36 species showed poor regeneration. Among these, 44.44% of them were less frequent, dense and dominant with IVI values < 1. According to Shibiru and Balcha [11], such species should be given priority for conservation. Species with high IVI values also require close monitoring as they are influential to the maintenance of a healthy ecosystem [16].

## 5. Conclusion

The assessment of woody plant species population structure and natural regeneration status was conducted in Tulu Korma afromontane forest. The findings of the study revealed the occurrence of 101 woody plant species distributed in 79 genera and 45 families in the studied site. Analysis of population structure based on all species combined densities showed that density of seedling > sapling > mature individuals, suggesting stable population structure and good regeneration status with an inverse J-shape pattern. Nevertheless, some species with different patterns of population structure displayed poor regeneration status. Hence, such species with least IVI and poor regeneration are in jeopardy and should be given priority for conservation. Thus, well-organized long term conservation approaches are endorsed for an improved forest management.

## Figures and Tables

**Table 1:** Density (no. of stems ha^−1^), frequency (%), basal area (dominance) (m^2^ ha^−1^) and important value index of woody plant species with DBH ≥ 3.5 cm.

No	Scientific name	Family	D	Fr	BA	IVI
1	*Acacia abyssinica* hocst. ex benth	Fabaceae	171	58	14.89	21.32
2	*Acacia albida* delile	Fabaceae	16	35	0.16	2.41
3	*∗Acacia mearnsii* De wild	Fabaceae	4	14	0.03	0.85
4	*Acacia melanoxylon* R. Br	Fabaceae	20	23	0.16	2.03
5	*Acacia seyal* Delile	Fabaceae	6	37	0.05	2.11
6	*Acanthus sennii* Chiov	Acanthaceae	37	19	0.05	1.87
7	*Adhatoda schimperiana* (Hochst.) Hochst. ex Nees	Acanthaceae	1.12	21	0.001	1.09
8	*∗Aeschynomene abyssinica* (A. Rich.) vatke	Fabaceae	15	6	0.04	0.82
9	*Albizia schimperiana* Oliv	Fabaceae	1	25	0.05	1.34
10	*Allophylus abyssinicus* (Hochst.) Radlk	Sapindaceae	32	10	0.07	1.67
11	*Apodytes dimidiata* E. Mey. ex Arn	Icacinaceae	39	38	0.04	3.23
12	*Arundo donax* L	Poaceae	25	40	0.07	2.89
13	*Bersama abyssinica* Fresen	Melanthiaceae	65	19	1.48	4.34
14	*Bougainvillea spectabilis* Willd	Nyctaginaceae	9	21	0.01	1.37
15	*Brucea antidysenterica* J.F. Mill	Simaroubaceae	30	25	1.07	3.16
16	*Buddleja davidii* Franch	Budlejaceae	5	13	0.003	0.82
17	*Buddleja polystachya* Fresen	Budlejaceae	1	17	0.001	0.89
18	*Caesalpinia decapetala* (Roth) Alston	Fabaceae	2	6	0.001	0.36
19	*Callistemon citrinus* (Curtis) Skeels	Myrtaceae	8	6	0.004	0.55
20	*Calpurnia aurea* (Aiton) Benth	Fabaceae	84	12	0.47	4.47
21	*∗Capparis tomentosa* Lam	Capparidaceae	8	13	0.05	0.94
22	*Carissa spinarum* L	Apocynaceae	106	52	5.34	10.67
23	*Cassipourea malosana* (Baker) Alston	Rhizophoraceae	1	15	0.001	0.78
24	*Clausena anisata* (Willd.) Hook.f. ex Benth	Rutaceae	1	17	0.01	0.90
25	*Coffea arabica* L	Rubiaceae	3	6	0.01	0.39
26	*Cordia africana* Lam	Boraginaceae	138	48	5.48	11.60
27	*Crotalaria rosenii* (Pax) Milne-Redh. ex Polhill	Fabaceae	4	6	0.03	0.45
28	*Croton macrostachyus* Hochst. ex Delile	Euphorbiaceae	100	46	5.44	10.26
29	*Cupressus lusitanica* Mill	Cupressaceae	3	25	0.02	1.36
30	*Dicrostachys cinerea* (L.) Wight & Am	Fabaceae	10	4	0.02	0.54
31	*Dodonaea angustifolia* L. f	Sapindaceae	97	10	0.19	3.83
32	*Dovyalis abyssinica* (A.Rich.) Warb	Flacourtiaceae	10	42	0.02	2.45
33	*Ekebergia capensis* Sparrm	Meliaceae	9	38	0.22	2.39
34	*Embelia schimperi* Vatke	Myrsinaceae	6	37	0.01	2.06
35	*∗Entada abyssinica* Steud. ex A.Rich	Fabaceae	3	10	0.01	0.61
36	*∗Erythrina brucei* Schweinf	Fabaceae	8	13	0.09	0.98
37	*Eucalyptus camaldulensis* Dehnh	Myrtaceae	23	8	0.16	1.25
38	*Eucalyptus globulus* Labill	Myrtaceae	6	12	0.02	0.80
39	*Euclea divinorum* Hiern	Ebenaceae	63	4	0.44	2.86
40	*Euphorbia abyssinica* J.F.Gmel	Euphorbiaceae	50	15	0.22	2.64
41	*Euphorbia pulcherrima* Willd. ex Klotzsch	Euphorbiaceae	16	13	0.02	1.18
42	*Ficus palmata* Forssk	Moraceae	139	8	1.63	6.34
43	*Ficus sur* Forssk	Moraceae	17	50	1.61	4.45
44	*∗Ficus sycomorus* L	Moraceae	7	6	0.22	0.71
45	*Ficus vasta* Forssk	Moraceae	6	4	0.80	1.08
46	*∗Flacourtia indica* (Burn.f.) Merr	Flacourtiaceae	7	7.56	0.01	0.45
47	*Galiniera saxifraga* (Hochst.) Bridson	Rubiaceae	38	6	0.06	1.59
48	*Grewia ferruginea* Hochst. ex A. Rich	Tiliaceae	33	4	0.24	1.47
49	*Hypericum quartinianum* A. Rich	Hypericaceae	11	23	0.01	1.51
50	*Juniperus procera* Hochst. ex Endle	Cupressaceae	180	42	12.16	18.46
51	*Laggera pterodonta* (DC.) Sch. Bip. ex Oliv	Asteraceae	5	21	0.003	1.22
52	*Lippia adoensis* var. *koseret* Sebsebe	Verbenaceae	6	12	0.01	1.16
53	*Maesa lanceolata* Forssk	Myrsinaceae	3	6	0.02	0.41
54	*∗Maytenus arbutifolia* (Hoscht. ex A. Rich.) R. Wilczek	Celastraceae	8	6	0.02	0.58
55	*∗Maytenus gracilipes* (Welw. ex Oliv.) Exell	Celastraceae	3	6	0.01	0.41
56	*∗Maytenus obscura* (A. Rich.) Cufod	Celastraceae	4	10	0.01	0.64
57	*Maytenus undata* (Thunb.) BlakeBlakelock	Celastraceae	5	8	0.01	0.55
58	*Millettia ferruginea* (Hochst.) Hochst. ex Baker	Fabaceae	45	13	1.10	3.07
59	*Myrica salicifolia* Hochst. ex A. Rich	Myricaceae	4	15	0.04	0.92
60	*Myrsine africana* L	Myrsinaceae	26	19	0.32	2.06
61	*Nuxia congesta* R.Br. ex Fresen	Loganiaceae	5	6	0.002	0.46
62	*Ocimum lamiifolium* Hochst. ex Benth	Lamiaceae	2	4	0.001	0.26
63	*Ocimum urticifolia* Roth	Lamiaceae	3	4	0.002	0.30
64	*Olea europaea* L	Oleaceae	247	63	29.99	37.06
65	*Osyris quadripartita* Salzm. ex Decne	Santalaceae	3	13	0.023	0.77
66	*Otostegia tomentosa* subsp. *ambigens* (Chiov.) Sebald	Lamiaceae	8	11	0.01	0.80
67	*Pavetta abyssinica* Fresen	Rubiaceae	4	10	0.001	0.61
68	*Phoenix reclinata* Jacq	Arecaceae	1	6	0.001	0.33
69	*Phytolacca dodecandra* L`Herit	Phytolaccaceae	1	17	0.001	0.88
70	*Pittosporum viridiflorum* Sims	Pittosporaceae	24	21	0.18	1.98
71	*Podocarpus falcatus* (Thunb.) C.N. Page	Podocarpaceae	173	67	25.90	31.32
72	*Premna schimperi* Engl	Verbenaceae	3	25	0.01	1.34
73	*∗Pterolobium stellatum* (Forssk.) Brenan	Fabaceae	7	4	0.01	0.42
74	*Rhamnus prinoides* L'Herit	Rhamnaceae	4	27	0.01	1.50
75	*Rhamnus staddo* A.Rich	Rhamnaceae	22	17	0.09	1.63
76	*Rhus glutinosa* Hoscht. ex A. Rich	Anacardiaceae	65	29	0.91	4.36
77	*Rhus vulgaris* Meikle	Anacardiaceae	10	21	0.12	1.48
78	*Ricinus communis* L	Euphorbiaceae	45	33	0.17	3.89
79	*∗Rosa abyssinica* R.Br. ex Lindl	Rosaceae	3	13	0.01	0.77
80	*Rubus steudneri* Schweinf	Rosaceae	37	31	0.12	2.86
81	*Rytigynia neglecta* (Hiern) Robyns	Rubiaceae	8	13	0.11	1.02
82	*Salix subserrata* Willd	Salicaceae	19	37	0.05	2.53
83	*Sapium ellipticum* (Hochst.) Pax	Euphorbiaceae	2	6	0.004	0.38
84	*Schefflera abyssinica* Harms	Araliaceae	49	34.6	0.15	3.14
85	*Schinus molle* L	Anacardiaceae	100	37	2.09	6.93
86	*Senna didymobotrya* (Fresen.) H.S. Irwin & Barneby	Fabaceae	7	9.62	0.01	0.71
87	*Sesbania sesban* (L.) Merr	Fabaceae	4	8	0.04	0.02
88	*∗Solanum incanum* L	Solanaceae	7	15	0.004	0.98
89	*Solanum marginatum* L.f	Solanaceae	90	15	0.11	3.79
90	*Stephania abyssinica* (Quart.-Dill. & A. Rich) Walp	Menispermaceae	10	33	0.06	2.03
91	*Syzygium guineense* (Wild.) DC	Myrtaceae	9	23	0.004	1.45
92	*Tacazzea apiculata* Oliv	Apocyanaceae	5	10	0.028	0.69
93	*Teclea nobilis* Delile	Rutaceae	11	10	0.13	0.97
94	*∗Triumfetta rhomboidea* Jacq	Tiliaceae	6	13	0.06	0.90
95	*Vepris dainelli* (Pic.Serm.) Kokwaro	Rutaceae	33	17	0.07	1.99
96	*Vernonia adoensis* Sch. Bip. ex Walp	Asteraceae	104	38	0.66	5.86
97	*Vernonia amygdalina* Delile	Asteraceae	35	21	0.04	2.22
98	*Vernonia leopoldii* (Sch. Bip.) Vatke	Asteraceae	14	13	0.20	1.28
99	*Vernonia myriantha* Hook.f	Asteraceae	1	6	0.001	0.33
100	*Vernonia urticifolia* A. Rich	Asteraceae	36	29	0.04	2.66
101	*Ximenia americana* L	Olacaceae	20	37	0.02	2.52
	Total		3020		116.18	299.03

*Note*. *D* = density, Fr = frequency, BA = basal area (dominance) and IVI = important value index. ∗ indicates species with poor regeneration status and least IVI (<1) values.

## Data Availability

The data used to support the study are included in the paper.
